# Roadmap to improve regional care for patients with severe asthma

**DOI:** 10.1002/clt2.12080

**Published:** 2021-12-02

**Authors:** J. P. M. van der Valk, J. H. Kappen, J. S. J. A. van Campen, G. Epping, J. M. A. M. Retera, D. de Bondt, F. Suwandy, M. Tolboom, J. C. C. M. in 't Veen, G. J. Braunstahl

**Affiliations:** ^1^ Department of Pulmonary Medicine STZ Center of Excellence for Asthma and COPD Franciscus Gasthuis & Vlietland Rotterdam The Netherlands; ^2^ Department of Pulmonary Medicine Haaglanden Medical Center The Hague The Netherlands; ^3^ Department of Pulmonary Medicine Elizabeth Twee‐Steden Hospital Tilburg The Netherlands; ^4^ AstraZeneca BV The Hague The Netherlands; ^5^ Vintura, Health Care Baarn The Netherlands; ^6^ Department of Pulmonary Medicine Erasmus Medical Center Rotterdam The Netherlands

1

To the Editor,

Severe asthma (SA) is a complex multifactorial disease, that is, often treated in a limited number of highly specialized care centers. Proper identification of all treatable traits is pivotal for an optimal personalized patient care plan. Regional collaboration in a “multidisciplinary consultation” (MDC) with pulmonologists, respiratory nurse, physiotherapist, and dietician is a tool to optimize this SA care.^1^ The MDC might contribute to the unmet referral need of asthma patients and potentially lead to a decrease in the number of hospital admissions and asthma exacerbations with subsequent improved quality of life.^2^, ^3^, ^4^, ^5^, ^6^

Furthermore, it will result in more empowerment of the local pulmonologist, better awareness of SA, better characterization of the “treatable traits” and in depth endotyping for appropriate biological indication. Hence, an optimal MDC follows the “right care, right place” paradigm focusing on the individual needs for the specific patient. The importance of a regional MDC is emphasized by the Dutch guideline for SA^7^ however, implementation of this infrastructure proves to be a challenge.

The Center of Excellence for SA (CEA) in South‐West Netherlands (Franciscus Gasthuis & Vlietland) has taken the initiative to design a roadmap to optimize the regional care for SA patients. The core team of the CEA consists of three pulmonologists, one respiratory nurse, and one physiotherapist. The regional collaboration consists of 12 hospitals, 1 tertiary center for high‐altitude pulmonary rehabilitation, and an extensive general practitioner network.

The first phase of the regional care project started by introducing the MDC in January 2019. Patients could be discussed in the MDC with difficult‐to‐treat asthma (uncontrolled asthma despite high‐dose inhaled corticosteroids and long‐acting beta‐2‐mimetics; GINA step 4–5 treatment) or SA (a subset of difficult‐to‐treat asthma with uncontrolled asthma despite maximally optimized “treatable traits” as for example therapy adherence, co‐morbidities, weight, and exercise).^7^

Until now, approximately 320 patients were assessed in weekly sessions (108 MDCs in total/2–4 patients per MDC). Patients were digitally presented by their own (local) pulmonologist using a standardized format: a personalized patient care plan was agreed by CEA consensus.

The second phase of the project was set up as a Delphi‐like procedure,^8^ in which health care professionals, pharmacists, insurance companies, patient associations, and patients were interviewed in order to include all perspectives in the roadmap. The outcomes of these interviews were discussed and analyzed in three subsequent work sessions resulting in the following goals^1^: regional standardization of SA care,^2^ identification and subsequent elimination of barriers for optimizing SA care and^3^ implementation of SA care in the region (Figure 1).

**FIGURE 1:**
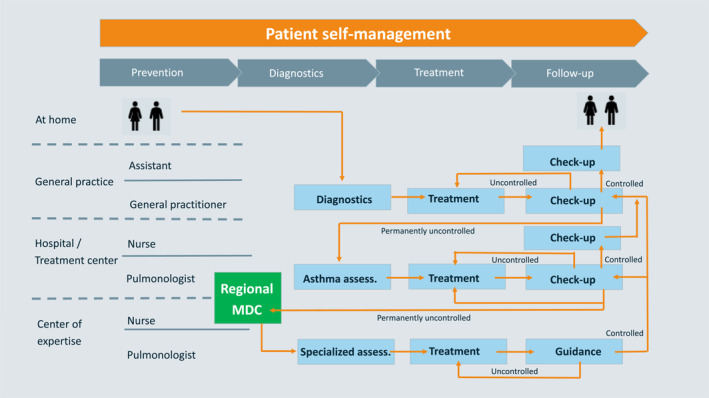
The optimal care pathway for severe asthma (SA) with the central role of the regional multidisciplinary consultation (MDC). This figure demonstrates the optimal SA care pathway with different phases of care: prevention, diagnostics, treatment, and follow‐up. The collaboration of the patient, general practitioner, hospital/treatment center, and the Center of Excellence for SA with a central role of the MDC is crucial in optimal SA care

Next a regional roadmap was designed in four strategic pillars to improve the care for SA patients in the region; organization of care, research, knowledge dissemination, and patient participation (Figure 2). Hence, the regional roadmap focusses on a uniform working process with a transmural SA care pathway, an integrated information technology and knowledge infrastructure, monitoring of care and appropriate funding of the regional MDC (Figure 3).

**FIGURE 2:**
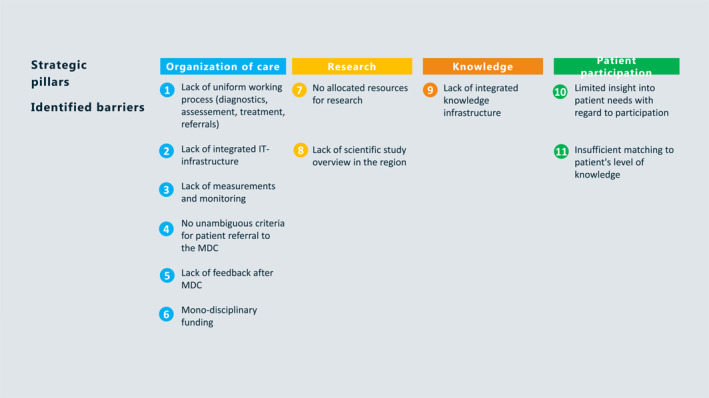
Strategic pillars and identified barriers for optimal care for severe asthma (SA) patients in the region. This figure shows the different strategic pillars; organization of care, research, knowledge and patient participation, and the identified barriers for optimal care for SA patients in the region

**FIGURE 3:**
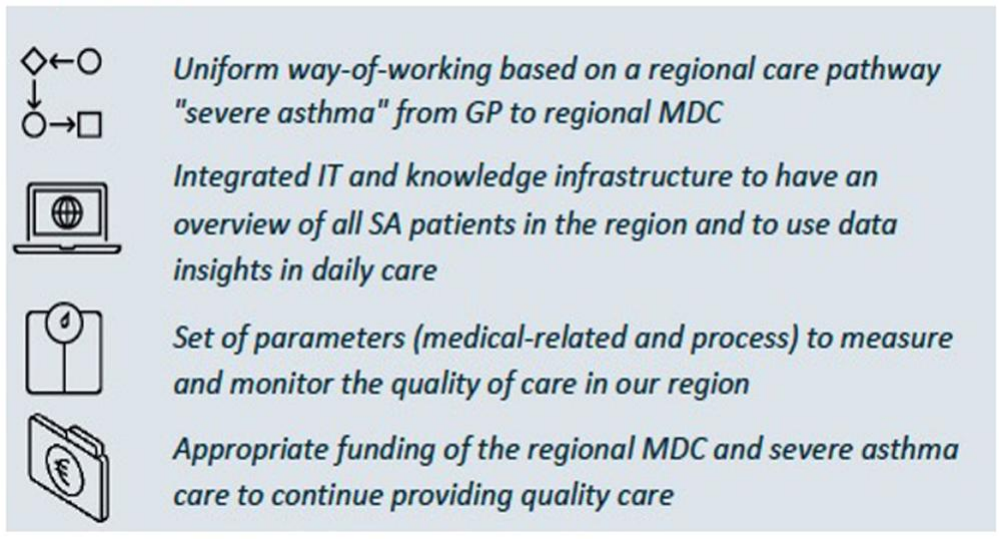
Requirements to improve regional severe asthma care

This project was a successful starting point to optimize SA care; the intervention will be further elaborated and implemented on a structured, project‐based approach to keep momentum and focus. The progress of this project will be evaluated monthly by the core team. The approach, experiences, and outcomes will be shared broadly within the region and nationally with pulmonologists, insurers, patient associations, and general practitioners specialized in SA care. We will endeavor for optimal MDC registration; to include all SA patient in the Registry of Adult Patients with Severe asthma for Optimal Disease management (RAPSODI) registry‐study. Patient data of SA, such as indication for biological treatment, the percentages SA patients with a personalized patient care plan, and the percentage of treatment centers with a pulmonologist specialized in SA care will be monitored.

The roadmap has the potential to improve SA care with the involvement of all stakeholders, a uniform work process in the MDC region and optimal personalized care. Furthermore, this roadmap can be used as practical guidance for the optimization and implementation of other SA MDC, national and/or international.

## CONFLICT OF INTEREST

The authors have no conflict of interest.

## FUNDING INFORMATION

AstraZeneca
